# One-Pot Synthesis and Immobilization of Gold Nanoparticles Using Peptidyl Microbeads

**DOI:** 10.3390/molecules30081689

**Published:** 2025-04-10

**Authors:** Shuhei Yoshida, Koki Yoshida, Taichi Isozaki, Maho Oura, Makoto Ozaki, Takaaki Tsuruoka, Kenji Usui

**Affiliations:** Faculty of Frontiers of Innovative Research in Science and Technology (FIRST), Konan University, 7-1-20 Minatojima-Minamimachi, Chuo-ku, Kobe 650-0047, Japan; d2361003@s.konan-u.ac.jp (S.Y.); isozaki411242@icloud.com (T.I.); s1591007@a.konan-u.ac.jp (M.O.); d1861001@a.konan-u.ac.jp (M.O.); tsuruoka@konan-u.ac.jp (T.T.)

**Keywords:** gold mineralization, mineralization, gold nanoparticle, peptide, immobilized peptide, gold-reducing peptide, immobilization

## Abstract

Gold nanoparticles (AuNPs) have surface plasmon resonance (SPR) and catalytic activity that are not found in bulk gold and have been studied in various fields. Among these, immobilization of AuNPs on various solid-phase substrates is known to produce stable catalytic activity and specific SPRs and research on the immobilization of AuNPs has been conducted actively. However, the conventional method requires the preparation and immobilization of AuNPs in separate processes, making it difficult to prepare immobilized AuNPs in a one-pot process. In this study, we attempted to synthesize and immobilize AuNPs using peptidyl beads, which are microbeads having immobilized a peptide capable of reducing gold ions. We successfully reduced Au ions from 0.5 to 1000 µM of HAuCl_4_ and immobilized them on peptidyl beads in the form of AuNPs. The immobilized AuNPs have a constant particle size independent of the HAuCl_4_ concentration. Furthermore, the peptidyl beads with AuNPs have catalytic activity. The quantity of the AuNPs on the peptidyl beads and, subsequently, the catalytic reaction rate of the sample, could be controlled. This study would also be expected to be applied to the immobilization of metallic nanomaterials other than AuNPs by modifying the peptide sequence.

## 1. Introduction

In recent years, research has been conducted to immobilize enzymes, complexes, and nanoparticles on solid-phase supports for molecular recognition and catalytic reactions [1,2,3,4]. Immobilization of molecules and nanoparticles prevents their aggregation and enables their reuse. Among these methods, the immobilization of metal nanoparticles has attracted considerable attention in recent years. Gold nanoparticles (AuNPs) have surface plasmon resonance (SPR), relative stability, and catalytic activity, and are actively being studied for application to sensing, probing, and catalytic materials [5,6,7]. Particularly, immobilization of AuNPs on various solid-phase supports has been reported [8,9,10]. For example, immobilized AuNPs on titania (TiO_2_) nanoparticles (Au-TiO_2_ nanocomposites) exhibit high photocatalytic activity [11]. However, conventional methods which are used to synthesize and immobilize AuNPs require at least two steps in the process. First, AuNPs are prepared. Next, chemical modification and calcination are used to immobilize the AuNPs on the solid phase. In other words, it is difficult to simultaneously prepare and immobilize AuNPs on the solid phase using a one-pot process. Optimization of the AuNP particle size and the fixation method should be considered separately. Therefore, we prepared and immobilized AuNPs using a one-pot process. Particularly, we focused on biomineralization using peptides. Peptides have been reported to selectively reduce metal ions in solution [12,13]. In the case of Au, it has been shown that AuNPs can be prepared in solution by mixing peptides and gold(III) chloride acid (HAuCl_4_) without any reducing agent [14,15,16]. Many of these peptides are known to utilize the π-electrons of the aromatic rings in their side chains such as tryptophan (Trp) and tyrosine (Tyr) to reduce gold ions to form AuNPs [17]. The particle size of the formed AuNPs can be controlled by changing in the peptide sequence the number of Trp residues involved in the reduction in Au ions [18]. In this report, an increasing number of Trp residues in the peptide increases the reducing ability, causing predominance of the nucleation reaction and the production of small gold nanoparticles. Furthermore, Au nanostructures of various shapes can be fabricated by modifying the sequence [19,20]. Therefore, peptide-based biomineralization can minutely control the formation of AuNPs with minimal environmental impact. Additionally, by using resins without a cleavable linker, immobilized peptides onto these resins are easily obtained by solid-phase synthesis and subsequent deprotection [21]. From these points of view, we focused on AuNPs, and demonstrated the synthesis of immobilized AuNPs by mineralization using microbeads having immobilized gold-reducing peptides. We hypothesized that by using the peptidyl beads gold ions could be reduced in situ, generating AuNPs that would therefore remain immobilized on the beads. Thus, we attempted to immobilize AuNPs easily on solid phase (the peptidyl beads) via a one-pot process without a protective and reducing agent. Furthermore, the initial catalytic reaction rate of the immobilized AuNPs was evaluated using the reduction of 4-nitrophenol as a model.

## 2. Results

### 2.1. Design and Synthesis of Au Ion-Reducing Peptide-Immobilized Beads

**Figure 1:**
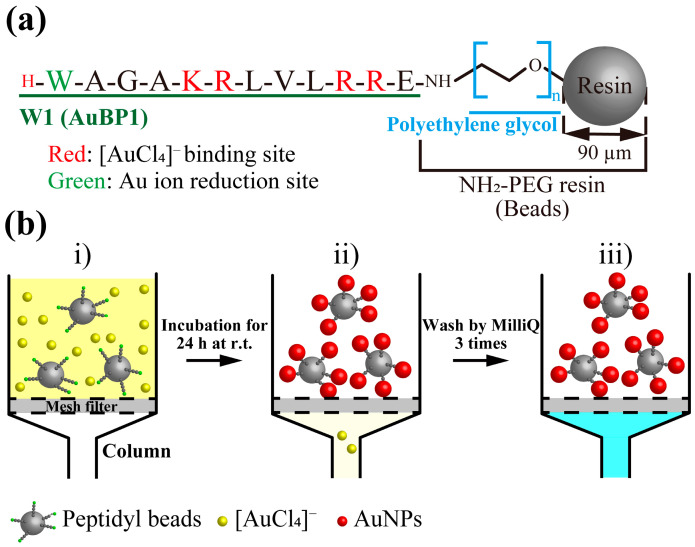
(**a**) Design and sequence of W1-beads. (**b**) Protocol of the preparation of immobilized AuNPs using peptidyl beads.

First, we selected a peptide sequence capable of reducing Au ions. AuBP1 (W1) has been shown to reduce Au ions to form AuNPs in previous studies [22]. The peptide was immobilized on aminoethyl polyethyleneglycol polystyrene microbeads (NH_2_-PEG resin) using direct Fmoc solid-phase synthesis [23] on NH_2_-PEG resin (beads). NH_2_-PEG resin is easily compatible with aqueous solvents because of the presence of PEG. In addition, a relatively low resin loading (the amount (mol) of amines per weight (g) of beads) was selected to ensure high purity of the peptides and to prevent aggregation of generated AuNPs. After solid-phase synthesis, deprotection was performed to obtain W1 immobilized on the beads (W1-beads, Figure 1a). The synthesized peptidyl beads were used after confirming their purity through amino acid analysis. Amino acid analysis showed that the loading of the peptidyl beads was 0.22 mmol/g. Acetylated NH_2_-PEG resin (Ac-beads) was prepared to investigate the effect of the microbeads on the reduction in Au ions. Additionally, W2-beads, in which the number of Trp residues at the N-terminus of W1 was changed to two, and W3-beads, in which the number was changed to three, were also designed. It has been reported that the particle size of AuNPs formed by these peptides in solution becomes smaller as the number of Trp increases [18]. Free W1 (Figure S1b) was also synthesized in the Fmoc solid phase using Fmoc-NH-SAL-PEG resin. Free W1 was deprotected, cleaved, and purified using high-performance liquid chromatography (HPLC, Figure S1c). After the purification, purity was confirmed using amino acid analysis.

### 2.2. Preparation of Immobilized AuNPs Using W1-Beads

#### 2.2.1. Preparation of Immobilized AuNPs

**Figure 2:**
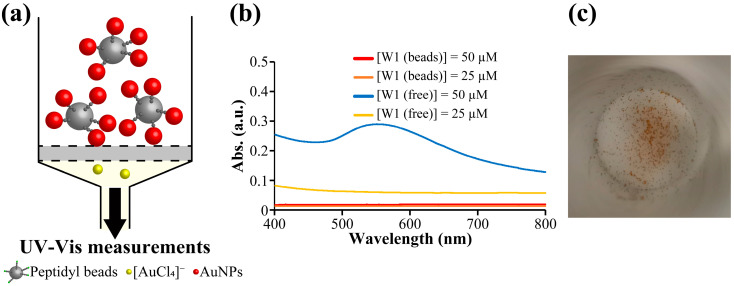
(**a**) UV-Vis measurements after the procedure in Figure 1b(ii). (**b**) Vis spectrum of the sample after Au mineralization using W1-beads and free W1. With the peptidyl beads, the filtered solution was used for UV-Vis measurements. (**c**) Images of W1-beads after Au ion reduction reaction with [W1 (beads)] = 25 µM. The reddish-black color shows W1-beads after the reaction.

We prepared AuNPs using W1-beads or W1 solution (free W1). In case of W1-beads, the procedure in Figure 1b was performed. AuNPs were prepared on W1-beads or with free W1 using [HAuCl_4_] = 100 µM and [W1 (beads)] or [W1 (free)] = 25 and 50 µM, which have been shown to form AuNPs in previous studies [18], and were compared using UV-Vis measurements (Figure 2). The concentration of W1-beads was calculated from the moles of peptide on the beads in the sample divided by the volume of the sample. With the free W1, SPR-derived absorbance of AuNPs was observed at approximately 520 nm, and the absorbance of 25 µM was much lower than that of 50 µM as previously shown in Ref. [18]. (Figure 2b). In contrast, no SPR-derived absorption of AuNPs was observed for using peptidyl beads (Figure 2b). This suggests that AuNPs formed by the reduction in Au ions using the peptide were retained on the W1-beads under [W1 (beads)] = 50 µM. In case of [W1 (beads)] = 25 µM, although UV-Vis results showed poor suggestion, it could be seen by the naked eye that the beads turned red (Figure 2c). This also suggests the beads also turned red in the [W1 (beads)] = 25 µM, suggesting the formation of AuNPs on the W1-beads. Therefore, we treated W1-beads and Ac-beads with aqua regia after the reaction with [HAuCl_4_] = 100 µM and [W1 (beads)] = 25 µM and quantified the amount of reduced Au ions using an inductively coupled plasma-atomic emission spectroscopy (ICP-AES) measurement. As a result, Au was detected under the condition of W1-beads and almost none under the condition of Ac-beads (Figure S2). Therefore, it is clear that Au ions were reduced on the W1-beads.

**Figure 3:**
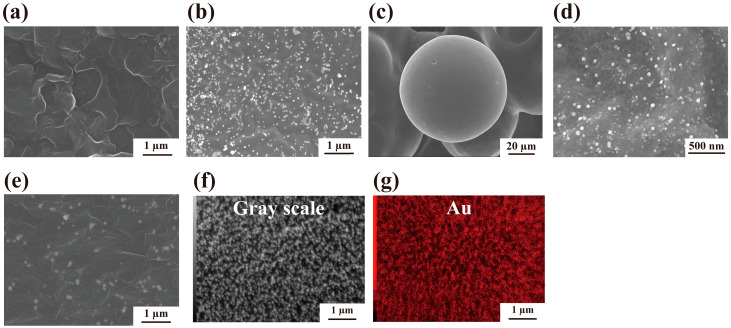
(**a**) SEM image of W1-beads before the Au ion reduction reaction. (**b**–**e**) SEM images of the sample after the Au ion reduction reaction using 25 µM of (**b**–**d**) W1-beads and (**e**) Ac-beads with 100 µM of HAuCl_4_. (**f**,**g**) SEM and SEM-EDX mapping images of the sample after the Au ion reduction reaction.

Furthermore, the shape of the reduced Au on the W1-beads after the reaction was observed using scanning electron microscopy (SEM). SEM observations showed that nothing was present on the W1-beads before the Au ion reduction (Figure 3a). However, the SEM results showed that spherical structures were formed on the W1-beads with [HAuCl_4_] = 100 µM and [W1 (beads)] = 25 µM (Figure 3b and Figure S3). To confirm that the W1-beads produce AuNPs in situ, a similar reaction was performed with Ac-beads. SEM observations showed that few spherical structures were formed on the Ac-beads (Figure 3c). In addition, W2-beads and W3-beads produced AuNPs with similar particle sizes to those of W1-beads (Figure S4). Furthermore, elemental analysis of the spherical structures formed on the W1-beads was confirmed from the mapping images and a spectrum using SEM-energy dispersive X-ray spectrometry (EDX, JEOL Ltd., Tokyo, Japan, Figure 3d,e and Figure S5). The spherical structures were identified as Au, indicating that the Au ions were reduced by W1-beads and immobilized as spherical AuNPs on the beads. These results showed that the W1-beads can reduce Au ions from the solution and immobilize them as site-specific AuNPs on the surface of W1-beads.

#### 2.2.2. Immobilization of AuNPs at Various Concentrations of W1 and HAuCl_4_

**Figure 4:**
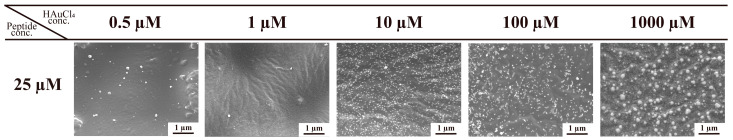
SEM images of the sample after the Au ion reduction reaction using 25 µM of W1-beads and 0.5–1000 µM of HAuCl_4_.

SEM was used to observe the shapes of the AuNPs formed at varying HAuCl_4_ concentrations. Reactions were performed using [W1(beads)] = 5, 10, and 25 µM with [HAuCl_4_] = 0.5–1000 µM. As a result, the formation of AuNPs was confirmed with [HAuCl_4_] = 1–1000 µM for [W1 (beads)] = 5, 10 µM and with [HAuCl_4_] = 0.5–1000 µM for [W1 (beads)] = 25 µM (Figure 4 and Figure S6). In other words, Au ions were reduced over a wide concentration range and immobilized on the W1-beads without aggregation or precipitation. Furthermore, the particle size of the immobilized AuNPs was confirmed to be as shown in Table 1, Figures S7–S10. With [W1 (beads)] = 25 µM, although the size distribution was broad, the particle size of the AuNPs was found to be approximately 25–50 nm (free W1 showed 15–30 nm as previous study Ref. [18]) and did not dynamically change in response to changes in the concentration of HAuCl_4_. In other words, the particle size did not change significantly despite changing the concentration of HAuCl_4_ in the solution. Previous studies have reported that several molecules of W1 were involved in the reduction reaction of Au ions [14]. However, because W1 was immobilized in this method, the reactivity was much lower than that in solution. Therefore, in this method it was suggested that the rate of reduction step would be limited, and the rate of Au ion reduction would not change significantly despite changing the concentration of HAuCl_4_ in the solution. These facts show that this method can produce AuNPs with similar particle sizes regardless of the concentrations of HAuCl_4_.

### 2.3. Evaluation of Initial Catalytic Reaction Rates of Immobilized AuNPs

We measured the initial catalytic reaction rates of the immobilized AuNPs on peptidyl beads (AuNP-W1-beads, AuNP-W2-beads, AuNP-W3-beads, and Ac-beads) in the reduction reaction of 4-nitrophenol to 4-aminophenol. The catalytic reaction rate was evaluated using AuNP-peptidyl beads prepared with [HAuCl_4_] = 100 µM, and [W1 (beads)], [W2 (beads)], [W3 (beads)], or [Ac (beads)] = 25 µM, and the reduction of 4-nitrophenol at [4-nitrophenol] = 125 µM, [NaBH_4_] = 10 mM, and [Tris-HCl] = 30 mM at room temperature. 4-Nitrophenol has a maximum absorption at 400 nm, and the absorption peak is decreased by reduction of 4-nitrophenol to 4-aminophenol by NaBH_4_. Therefore, the reaction rate can be calculated from this decrease in absorption intensity at 400 nm using UV-Vis measurement. The results showed that AuNPs prepared using all peptidyl beads were catalytically active (Figure S11). As a result, the samples of AuNPs-W1-beads, AuNPs-W2-beads, and AuNPs-W3-beads had comparable initial catalytic reaction rates, and they had higher rates than Ac-beads. The reduction reaction of 4-nitrophenol did not proceed with W1-beads and Ac-beads unreacted with Au ions (Figure S12). These results showed that immobilized AuNPs on peptidyl beads have catalytic activity.

**Figure 5:**
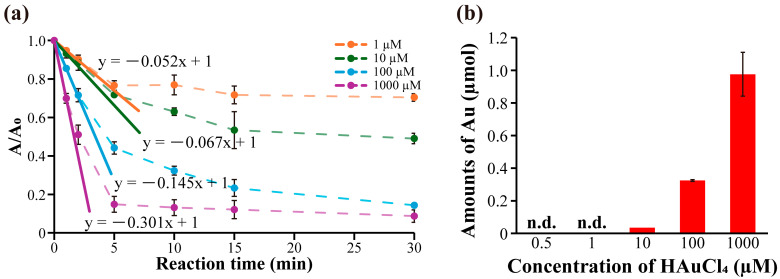
(**a**) Catalytic reaction progression of immobilized AuNPs prepared using W1-beads. Progression in immobilized AuNPs prepared under various conditions from 0 to 30 min. (**b**) Quantitative analysis of Au immobilized on W1-beads using ICP-AES measurement. n.d.: Detected values were less than 0.01 µmol.

Next, the initial catalytic reaction rates of the samples were evaluated by changing the concentration of HAuCl_4_ using W1-beads. In detail, AuNP-W1-beads were prepared at [HAuCl_4_] = 0.5–1000 µM, [W1 (beads)] = 25 µM, and the reduction was conducted with 4-nitrophenol at 125 µM and NaBH_4_ at 10 mM in 30 mM Tris-HCl at room temperature. The initial reaction rate was calculated from the amount of change in absorbance at 400 nm during the first 60 s of the reduction reaction. The initial reaction rates were −0.052 min^−1^ at [HAuCl_4_] = 1 µM, −0.067 min^−1^ at [HAuCl_4_] = 10 µM, −0.145 min^−1^ at [HAuCl_4_] = 100 µM, and −0.301 min^−1^ at [HAuCl_4_] = 1000 µM (Figure 5a and Figure S13). In other words, AuNP-W1-beads prepared at higher HAuCl_4_ concentrations showed faster initial catalytic reaction rates. The SEM observation results indicated that the particle size of the AuNPs prepared using this method was uniform. However, it has been reported that changes in catalytic activity are caused by the particle size and shape of the AuNPs [24]. Therefore, the difference in the initial catalytic reaction rates can be attributed to the amount of AuNPs fixed owing to the difference in the amount of Au ions. Reactions were conducted with [HAuCl_4_] = 100, 1000 µM and [W1 (beads)] = 25 µM, and the amount of Au immobilized on the beads was quantified using ICP-AES measurements. The results showed that the amounts of Au were immobilized 0.98 ± 0.120 µmol at [HAuCl_4_] = 1000, 0.32 ± 0.05 µmol at [HAuCl_4_] = 100 µM and 0.04 µmol at [HAuCl_4_] = 10 µM (Figure 5b). On the other hand, the amounts of immobilized AuNPs prepared at [HAuCl_4_] = 0.5 and 1 µM were below the limit of quantification for ICP-AES measurements. However, Au was detected, indicating the presence of less than 0.01 µmol of Au. At least [HAuCl_4_] = 1–1000 µM, the amount of Au reduction increases with increasing HAuCl_4_ concentration. In other words, the initial catalytic reaction rates of the samples increased proportionally with the amount of immobilized AuNPs. Additionally, the number of AuNPs in the sample was calculated by dividing the amount of Au atoms measured by ICP-AES (mol, Figure 5b) by the amount of Au atoms in one AuNP (mol, detailed calculation was described in Section 3.6). This implied that the number of AuNPs on the sample was positively correlated with the concentration of HAuCl_4_ (Figure S14). In summary, the number of AuNPs immobilized on the W1-beads increases with increasing HAuCl_4_ concentration and then correspondingly, the catalytic rates increased. These results show that catalytic rates of an immobilized AuNP sample prepared using this method with W1-beads can be adjusted by varying the concentration of HAuCl_4_.

## 3. Materials and Methods

### 3.1. General Remarks

All chemicals and solvents were of reagent or high-performance liquid chromatography (HPLC) grade and were used as received without further purification.

### 3.2. Synthesis of Peptidyl Beads and Peptide

Peptides were synthesized and immobilized onto NH_2_-PEG resin (0.26 mmol/g, Watanabe Chemical Industries Ltd. (Watanabe), Hiroshima, Japan) and were manually synthesized by the traditional 9-fluorenylmethoxycarbonyl (Fmoc)-based solid-phase peptide synthesis using Fmoc-NH-SAL-PEG resin (0.23 mmol/g, Watanabe). Peptide bonds were formed using 2-(1H-benzotriazole-1-yl)-1,1,3,3-tetramethyluronium hexafluorophosphate (HBTU, 10 eq., Watanabe) and 1-hydroxybenzotriazole monohydrate (HOBt, 10 eq., Watanabe) as a coupling reagent in the presence of Hunig’s Base (*N*,*N*-diisopropylethylamine, DIEA, 15 eq., Watanabe) for 30 min at 37 °C. The Au^3+^ reducing peptide sequences (H-WAGAKRLVLRRE-beads) were synthesized and immobilized using Fmoc-amino acids (Watanabe). The side-chain-protecting groups used were tert-butoxycarbonyl for tryptophan and lysine, 2,2,4,6,7-pentamethyl-2,3-dihydrobenzofuran-5-yl) sulfonyl for arginine, and tert-butoxy for glutamic acid. The side-chain-protecting groups on the peptidyl beads were removed by incubating the peptide beads for 2 h in a deprotection solution [trifluoroacetic acid (TFA, Watanabe)/triisopropylsilane (FUJIFILM Wako Pure Chemical Industries, Ltd. (Wako), Osaka, Japan)/thioanisole (Wako)/MilliQ (Milli-Q Reference, Merck KGaA (Merck), Darmstadt, Germany) (50:1:1:1, *v*/*v*/*v*/*v*)]. The beads were washed five times with a deprotection solution, MilliQ/0.1% TFA, and 1-methyl-2-pyrrolidone (NMP, Wako). The beads were then washed five times with chloroform (Wako) and completely dried in a desiccator. The peptide loading, peptide purity, and amino acid content of each peptide were evaluated using amino acid analysis. Amino acid analysis was performed using a COSMOSIL 5C_18_-AR-II column (4.6 × 250 mm; Nacalai Tesque, Inc., Kyoto, Japan) after samples were hydrolyzed in 6 M HCl (Wako) at 110 °C for 96 h in a sealed tube and then labeled with phenyl isothiocyanate (Wako). The same sequences were synthesized in parallel under the same conditions using Fmoc-NH-SAL-PEG resin (Watanabe). The respective purities were checked by HPLC [GL-7400 HPLC system (GL Sciences, Tokyo, Japan)] using an Inertsil ODS-3 column (4.6 × 150 mm; GL Science) with 0.1% TFA in MilliQ (A solution) and 0.08% TFA in an acetonitrile (Kanto Chemical Co., Inc., Tokyo, Japan, B solution) gradient system, at a flow rate of 1.0 mL/min, after the cleavage [TFA (Watanabe)/triisopropylsilane (Wako)/thioanisole (Wako)/MilliQ (50:1:1:1, *v*/*v*/*v*/*v*)] for 1 h. The peptides were analyzed using MALDI-TOF MS on an Axima Performance (Shimadzu Corporation, Kyoto, Japan) mass spectrometer with sinapic acid (Wako) as the matrix. Additionally, free W1 peptide was obtained by HPLC purification of the crude after the cleavage (Figure S1).

### 3.3. Synthesis of Ac-Beads

Ac-beads were obtained by acetylation of the NH_2_-PEG resin. First, NH_2_-PEG resin was swollen with NMP for 24 h and washed five times. Then, 1 mL of NMP was added, followed by the addition of 20 equivalents of acetic anhydride (Wako) and 10 equivalents of DIEA relative to the number of amino groups, and the reaction was conducted for 30 min at 37 °C. The reaction was performed in duplicate. The beads were washed with chloroform and vacuum-dried for 1 h. The dried beads were stored at 4 °C.

### 3.4. Reduction in Au Ions and Immobilization of AuNPs Using Peptidyl Beads or Free Peptide

In case of using the peptidyl beads, these were weighed on the column to obtain a peptide concentration of 5–25 µM when the total volume was 5 mL. (The concentration of W1-beads was calculated from the moles of peptide on the beads in the sample divided by the volume of the sample.) The peptidyl beads were weighed on PD-10 columns (Cytiva, Tokyo, Japan). The peptidyl beads were swollen in NMP for 24 h and then washed five times with methanol and MilliQ water before use. The reactions were conducted at room temperature with stirring at 1500 rpm using CM-1000 (Tokyo Rikakikai Co. Ltd., Tokyo, Japan). The reaction solution was mixed with MilliQ water up to 5 mL to obtain a concentration of 0.5–1000 µM HAuCl_4_ (Merck). After the reaction, the solution was removed and washed three times with 1 mL of MilliQ water.

In the case of free W1, in 1.5 mL microtubes, the HAuCl_4_ concentration was 100 µM, the W1 (free) concentration was 25 or –50 µM, and the total volume was 1 mL of MilliQ, and the reaction was allowed to run for 24 h at room temperature.

### 3.5. UV-Vis Measurement After Au Ion Reduction Reaction

UV-Vis spectroscopy was performed using a UV-1800 spectrometer (Shimadzu Corporation) by adding 130 μL of the post-reaction sample to an eight-cell micromulticell with a 1 cm pathlength.

### 3.6. Scanning Electron Microscopy (SEM), SEM-Energy Dispersive X-Ray Spectroscopy (SEM-EDX) Measurement, and Particle Size Analysis

After the reduction reaction of Au ions using the beads, the samples were vacuum-dried for 24 h after the PD-10 column filter was removed from the container. The beads were then placed on a SEM sample stand with an attached carbon tape (Nisshin-EM, Tokyo, Japan). An osmium coating (Neoc-Pro/P, Meiwafosis Co., Ltd., Tokyo, Japan) was applied to the SEM sample table on which the filter was placed. Shape observation using SEM and elemental analysis using SEM-energy dispersive x-ray spectroscopy (EDX) were performed using a JSM-7001FA instrument (JEOL Ltd., Tokyo, Japan) at an acceleration voltage of 15 kV. Particle size was determined by measuring 100 particles using ImageJ (ver. 1.54g) [25]. The number of AuNPs was calculated using the following formula.

$$\frac{4}{3}\times 3.14\times {\left(Particle\;size\;\left(nm,\;Table\;1\right)÷2\right)}^{3}=Volume\;of\;AuNPs\;\left({nm}^{3}\right)\ldots i$$

$$\frac{4}{3}\times 3.14\times {\left(1.44\;(Å,\;Theoritical\;value\;of\;atomic\;radius\;Of\;Au)\right)}^{3}\;\;=Theoritical\;volume\;of\;a\;single\;Au\;atom\;\left({nm}^{3}\right)\ldots ii$$

$$\frac{Volume\;of\;AuNPs\;\left(i\right)}{Theoretical\;volume\;of\;a\;single\;Au\;atom\;\left(ii\right)}=Number\;of\;Au\;atoms\;per\;AuNPs\ldots iii$$

$$(mount\;of\;immobilized\;Au\;obtained\;by\;ICP\;measurement\;\left(mol\right))\;\;\times Avogadro\;constant\;\left(6.02\times 10^{23}\;{mol}^{-1}\right)\;\;=Number\;of\;Au\;atoms\;immobilized\;on\;peptidyl\;beads\ldots iv$$

$$\frac{Number\;of\;Au\;atoms\;immobilized\;on\;peptidyl\;beads\;(iv)}{Number\;of\;Au\;atoms\;per\;AuNPs\;(iii)}=Number\;of\;AuNPs\;in\;the\;sample$$

### 3.7. Inductively Coupled Plasma-Atomic Emission Spectroscopy Measurement

After the reduction in the Au ions, the sample solution was removed and washed three times with 1 mL of MilliQ water. Then, 5 mL of (1 + 1) aqua regia was added to the washed W1-beads and stirred at 1500 rpm for 30 min to dissolve the Au nanoparticles formed on the W1-beads. The samples were pyrolyzed with 5 mL of HClO_4_ (Wako) for 1 h at 250 °C. After pyrolysis, the samples were dissolved in 2.5 mL of (1 + 1) aqua regia and 22.5 mL of MilliQ water. Calibration lines for each element were obtained using gold standard solution (for Atomic Absorption Spectrochemical Analysis, Wako) in the range from 0 to 10 ppm. Gold was detected at a wavelength of 242.795 nm using inductively coupled plasma-atomic emission spectroscopy (ICP-AES, SPECTROBLUE^®^FMX36, Hitachi, Tokyo, Japan). When ICP-AES measurements were performed using this technique, Au below 10 µM in the preparation amount was below the detection limit.

### 3.8. Initial Catalytic Reaction Rates Evaluation of Immobilized AuNPs Using the Reduction of 4-Nitrophenol

The immobilized AuNPs were washed three times with 1 mL of MilliQ water and used for catalytic reactions. Then, 2.5 mL of 20 mM NaBH_4_ (Wako) was added to the immobilized AuNPs and incubated at 1500 rpm at room temperature for 1 min. After incubation, 125 µL of 5 mM 4-nitrophenol (Sigma-Aldrich, St. Louis, MO, USA) and 750 µL of 0.2 M Tris-HCl buffer (pH 8.5, Wako) were added to this mixed solution and catalyzed at room temperature with stirring at 1500 rpm. Subsequently, 117 µL of MilliQ was added to 13 µL of the solution after the catalytic reaction and diluted 10-fold, and UV-Vis spectra of the materials were acquired using UV-1800. The initial catalytic reaction rates were evaluated by checking the absorbance at 400 nm derived from the reduction reaction of 4-nitrophenol.

## 4. Conclusions

In conclusion, we successfully immobilized AuNPs easily on solid phase via a one-pot process using peptidyl beads without a protective and reducing agent. Furthermore, the immobilized AuNPs were catalytically active. Immobilized AuNPs prepared using peptidyl beads were shown to have a constant particle size, regardless of changes in the HAuCl_4_ concentration. This suggests that this method can be used to prepare immobilized AuNPs with a constant particle size, independent of the concentration of Au ions in the solution, by controlling the affinity of the peptide for Au. Additionally, the amount of immobilized peptide might be controlled by changing the loading of beads in this method. Therefore, it is expected that the amount and particle size of the AuNPs formed could be controlled. Furthermore, the initial catalytic reaction rates of the immobilized AuNPs was found to increase in proportion to the concentration of HAuCl_4_, i.e., the amount of immobilized AuNPs, allowing a concentration-dependent control of the initial catalytic reaction rates. In general, morphology changes due to changing in the concentration of Au ions, and it is not easy to change only the number of particles. This is a rare report of successful control of catalytic amount by controlling the amount of AuNPs with the same morphology according to the concentration of Au ions. Moreover, this method would easily immobilize metal nanoparticles other than Au such as Ag and Cu by changing the peptide to other metal-specific reduction sequences [26,27]. Therefore, it is expected to be an easy one-pot method for synthesizing various nanostructures of two or more metals by their concentrations.

## Figures and Tables

**Table 1:** Particle size of AuNPs calculated from the histograms (Figures S8–S10).

		Concentration of HAuCl_4_				
		0.5 µM	1 µM	10 µM	100 µM	1000 µM
**Concentration of peptide**	**5 µM**	n.d.	70.6 ± 54.5 nm	37.3 ± 13.7 nm	85.7 ± 47.0 nm	38.2 ± 32.1 nm
	**10 µM**	n.d.	43.2 ± 15.8 nm	47.8 ± 14.1 nm	106.0 ± 27.0 nm	61.9 ± 25.8 nm
	**25 µM**	26.9 ± 6.6 nm	26.3 ± 13.9 nm	37.7 ± 11.5 nm	39.4 ± 12.6 nm	46.0 ± 35.3 nm

## Data Availability

The raw data supporting the conclusions of this article will be made available by the authors on request.

## Supplementary Materials

The following supporting information can be downloaded at https://www.mdpi.com/article/10.3390/molecules30081689/s1. Figure S1: Sequence of peptides and HPLC chromatogram of purified free W1; Figure S2: Quantitative analysis of immobilized Au using ICP-AES; Figure S3: SEM images of the sample after Au reduction reaction using W1-Beads; Figure S4: SEM images of the sample after Au reduction reaction using W2-beads and W3-beads.; Figure S5: EDX spectra of the sample after the Au ion reduction reaction using W1-beads; Figure S6: SEM images of the sample after Au reduction using W1-beads; Figure S7: Graph of particle size of immobilized AuNPs; Figure S8: Particle size histogram of the sample after gold reduction with [W1 (beads)] = 5 µM; Figure S9: Particle size histogram of the sample after gold reduction with [W1 (beads)] = 10 µM; Figure S10: Particle size histogram of the sample after gold reduction with [W1 (beads)] = 25 µM; Figure S11: Catalytic reaction progression of immobilized AuNPs prepared using peptidyl beads; Figure S12: Catalytic reaction progression of immobilized AuNPs prepared using control beads; Figure S13: Initial reaction progression of a catalytic reaction; Figure S14: The amounts of immobilized AuNPs on peptidyl beads.
