# Supplementary material for: One-Pot Synthesis and Immobilization of Gold Nanoparticles Using Peptidyl Microbeads
Source: Molecules. 2025 Apr 10;30(8):1689. doi: 10.3390/molecules30081689 (PMC12029603; doi:10.3390/molecules30081689)
Supplement: Supplementary file 1 [file molecules-30-01689-s001.zip › molecules-3508885-supplementary.pdf]

Supporting Information for

One-pot synthesis and immobilization of gold nanoparticles using peptidyl microbeads

*Shuhei Yoshida, Koki Yoshida, Taichi Isozaki, Maho Oura, Makoto Ozaki, Takaaki Tsuruoka and Kenji Usui**

Faculty of Frontiers of Innovative Research in Science and Technology (FIRST), Konan University, 7-1-20 Minatojima-Minamimachi, Chuo-ku, Kobe 650-0047, Japan

*Correspondence: kusui@konan-u.ac.jp; Tel.: +81-78-303-1418

CONTENTS:

Supplementary Figures:

Fig. S1 Sequence of peptides and HPLC chromatogram of purified free W1.

Fig. S2 Quantitative analysis of immobilized Au using ICP-AES.

Fig. S3 SEM images of the samples after Au reduction reaction using W1-Beads.

Fig. S4 SEM images of the sample after Au reduction reaction using W2-beads and W3-beads.

Fig. S5 EDX spectra of the sample after the Au ion reduction reaction using W1-beads.

Fig. S6 SEM images of the sample after Au reduction using W1-beads.

Fig. S7 Graph of particle size of immobilized AuNPs.

Fig. S8 Particle size histogram of the sample after gold reduction with [W1 (beads)] = 5 μM.

Fig. S9 Particle size histogram of the sample after gold reduction with [W1 (beads)] = 10 μM.

Fig. S10 Particle size histogram of the sample after gold reduction with [W1 (beads)] = 25 μM.

Fig. S11 Catalytic reaction progression of immobilized AuNPs prepared using peptidyl beads.

Fig. S12 Catalytic reaction progression of immobilized AuNPs prepared using control beads.

Fig. S13 Initial reaction progression of a catalytic reaction.

Fig. S14 The amounts of immobilized AuNPs on peptidyl beads.

Supplementary Figures

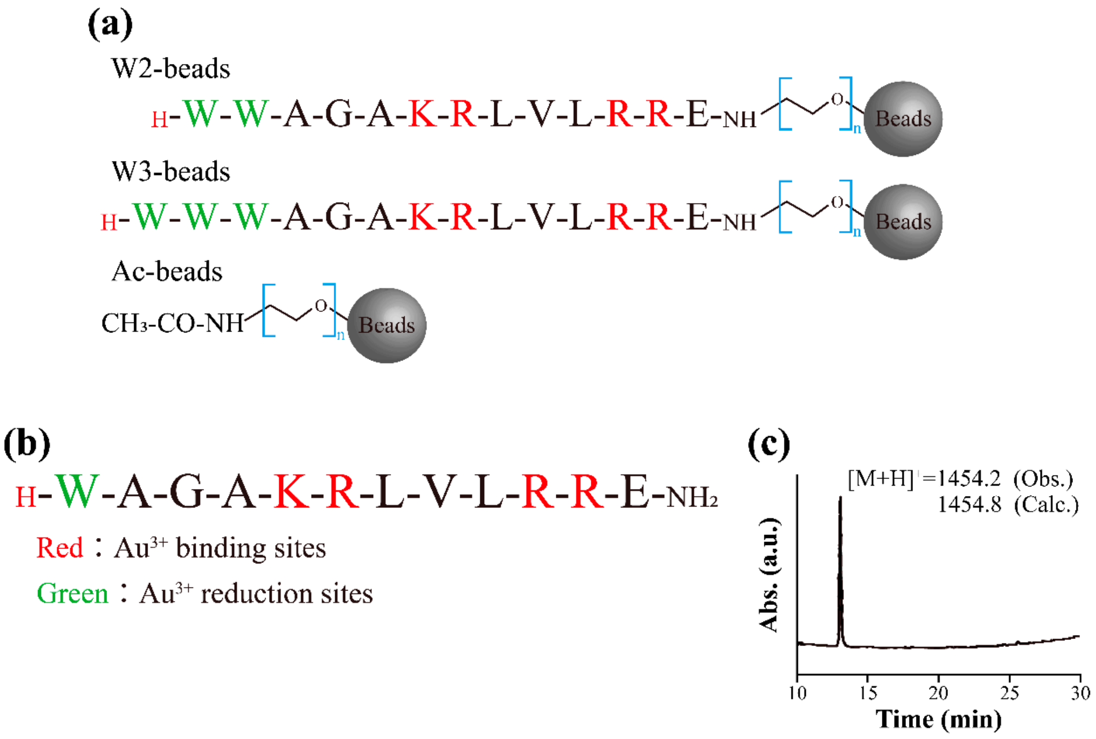

Fig. S1 (a) Design and sequence of W2-beads, W3-beads and Ac-beads. (b) Sequence of free W1. (c) HPLC chromatogram for purified W1 separated on an ODS column (150×4.6 mm, 5 μm) with MilliQ water (containing 0.1% TFA) using a gradient from 0% to 100% acetonitrile (containing 0.08% TFA) over 30 min, 1.0 mL/min; detection wavelength at 220 nm.

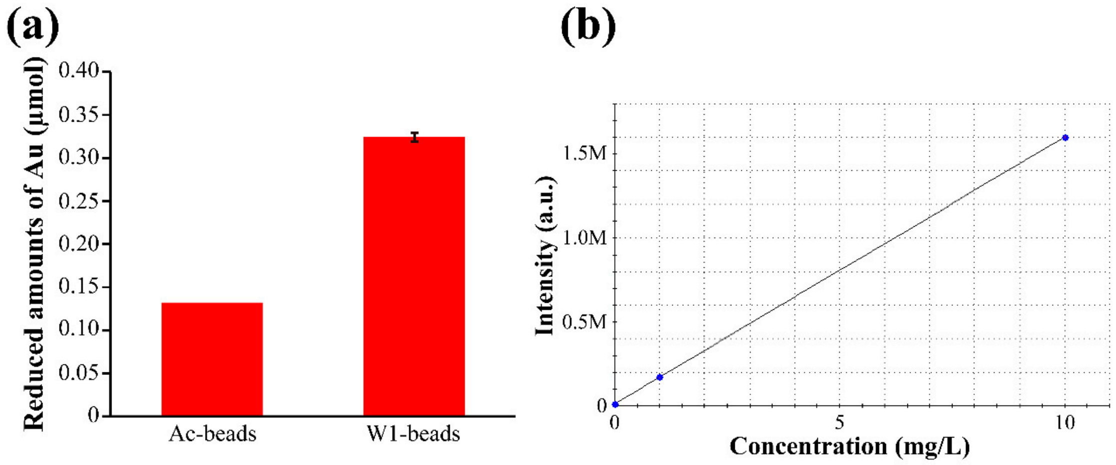

Fig. S2 (a) Quantitative analysis of Au immobilized on W1-beads or Ac-beads using ICP-AES measurement. The samples were prepared with [$HAuCl_4$] = 100 μM, [W1 (beads)] or [Ac (beads)] = 25 μM. (b) Calibration line prepared using 0, 1, and 10 ppm of Au standard solutions in ICP-AES measurements.

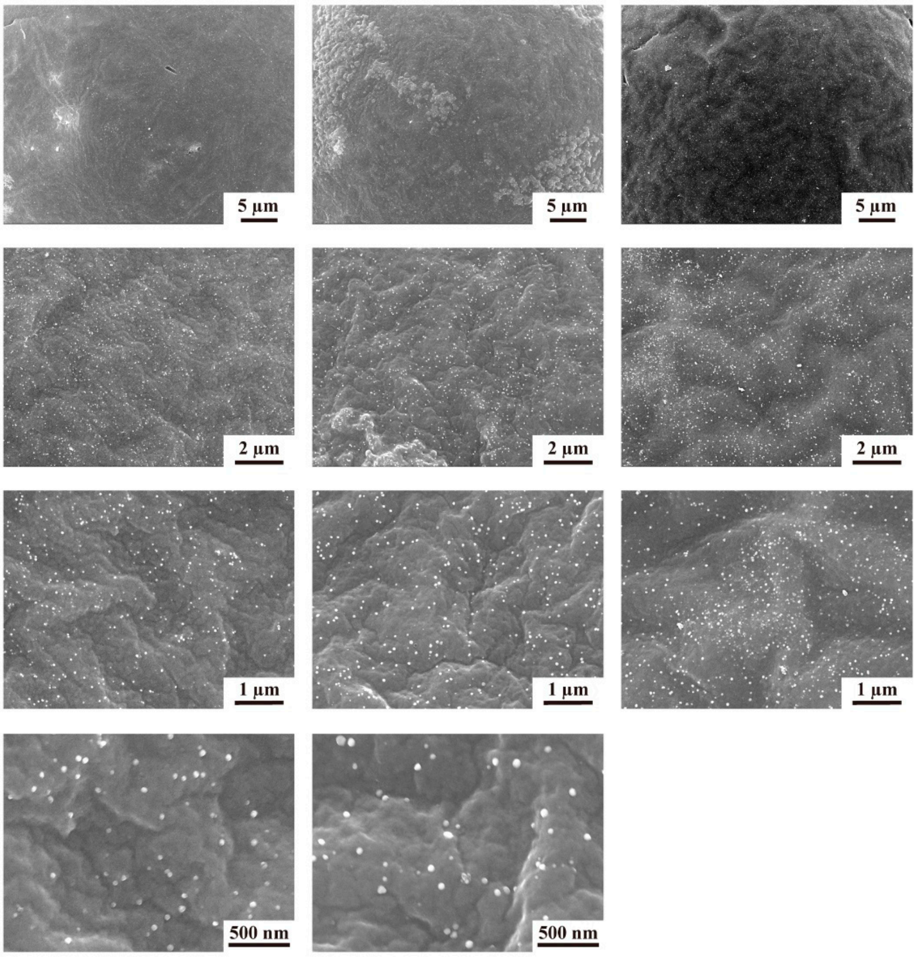

Fig. S3 SEM images with various magnification of the samples after Au reduction reaction using W1-Beads. [W1 (beads)] = 25 µM, [$HAuCl_4$] = 100 µM.

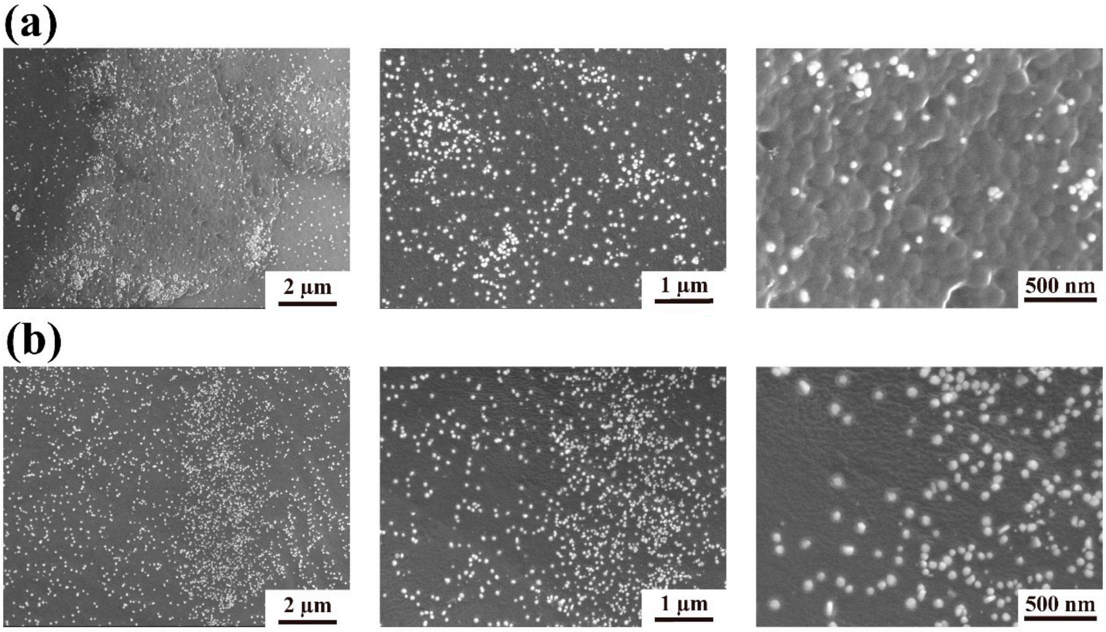

Fig. S4 SEM images of the sample after Au reduction reaction using (a) W2-beads and (b) W3-beads ([W2 (beads)] or [W3 (beads)] = 25 µM, [$HAuCl_4$] = 100 µM).

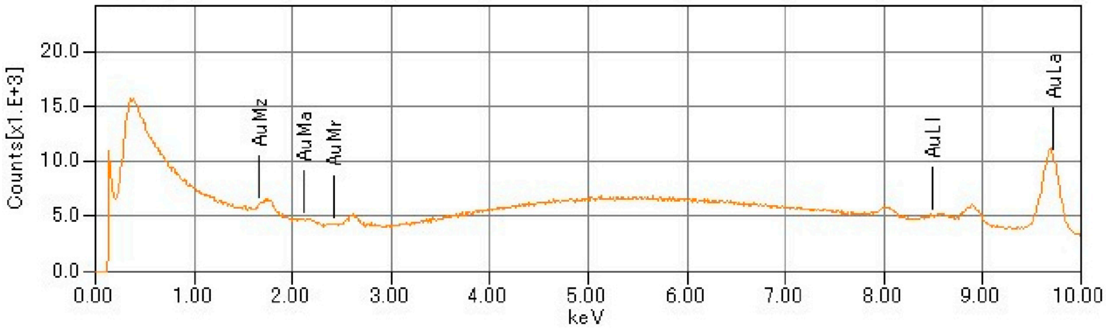

Fig. S5 EDX spectra of the sample after the Au ion reduction reaction using W1-beads. [W1 (beads)] = 25 μM, [$HAuCl_4$] = 100 μM.

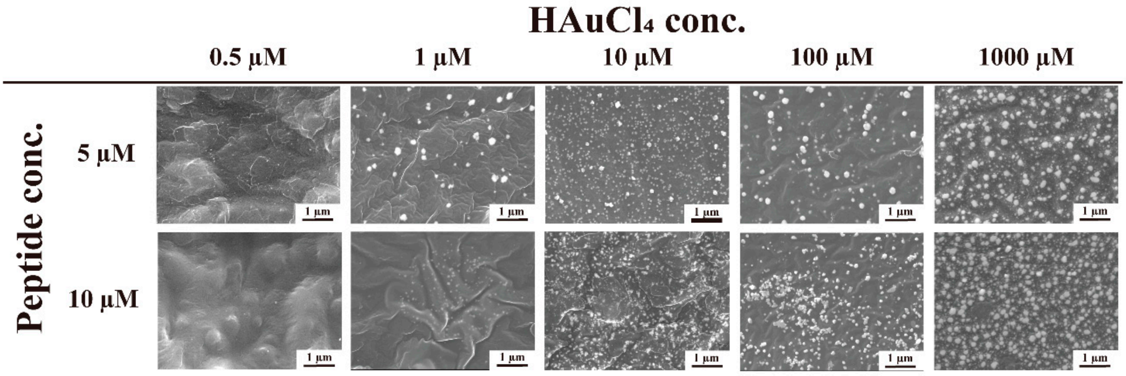

Fig. S6 SEM images of the sample after the Au ion reduction reaction using 5 or 10 μM scale of W1-beads and 0.5–1000 μM of $HAuCl_4$.

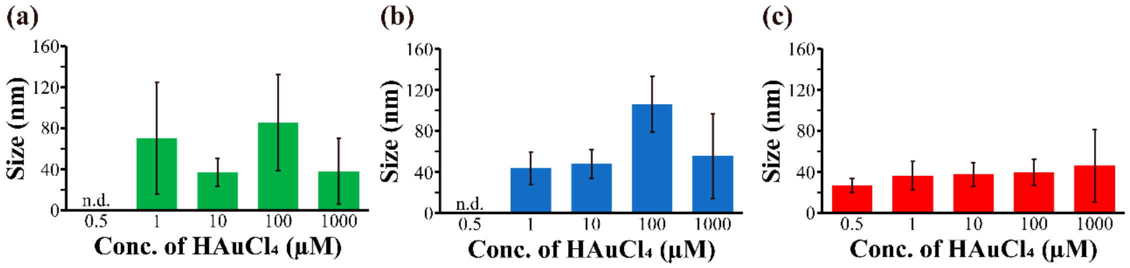

Fig. S7 (a-c) Particle size of immobilized AuNPs obtained from SEM images (N=100). (a, 5 μM W1; b, 10 μM W1; c, 25 μM W1)

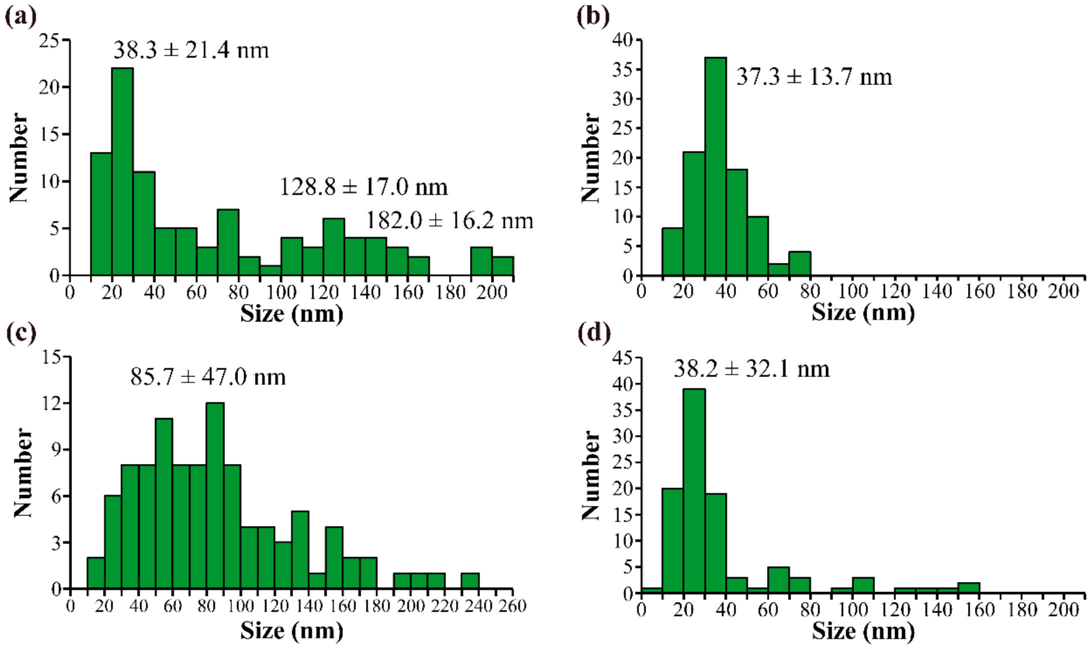

Fig. S8 Particle size histograms of the samples after gold reduction by using [W1 (beads)] = 5 μM with [$HAuCl_4$] = (a) 1 μM, (b) 10 μM, (c) 100 μM and (d) 1000 μM.

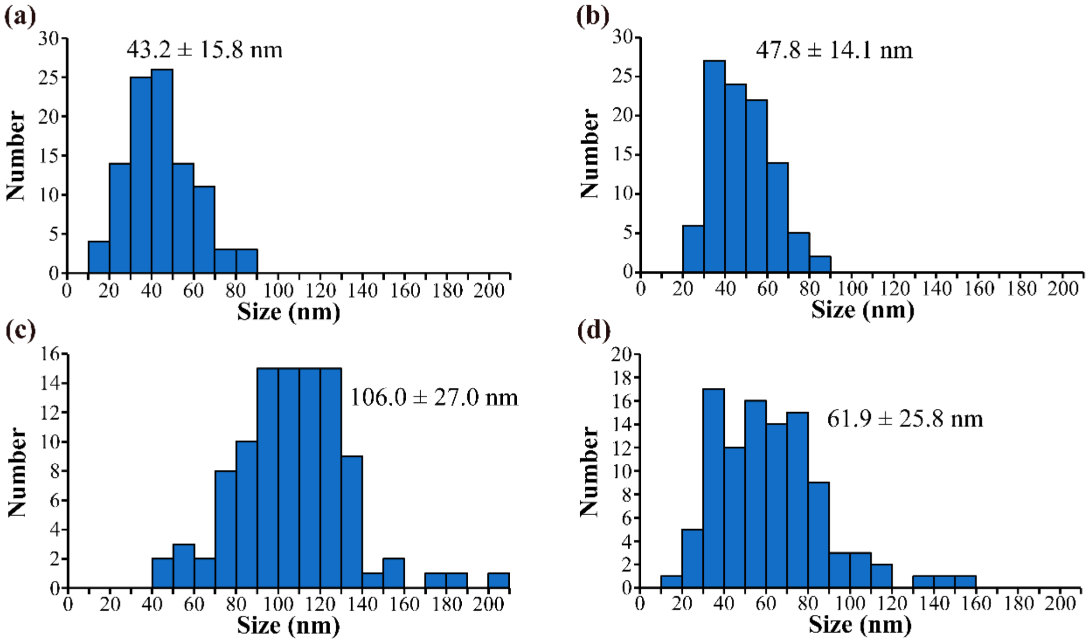

Fig. S9 Particle size histograms of the samples after gold reduction by using [W1 (beads)] = 10 μM with [$HAuCl_4$] = (a) 1 μM, (b) 10 μM, (c) 100 μM and (d) 1000 μM.

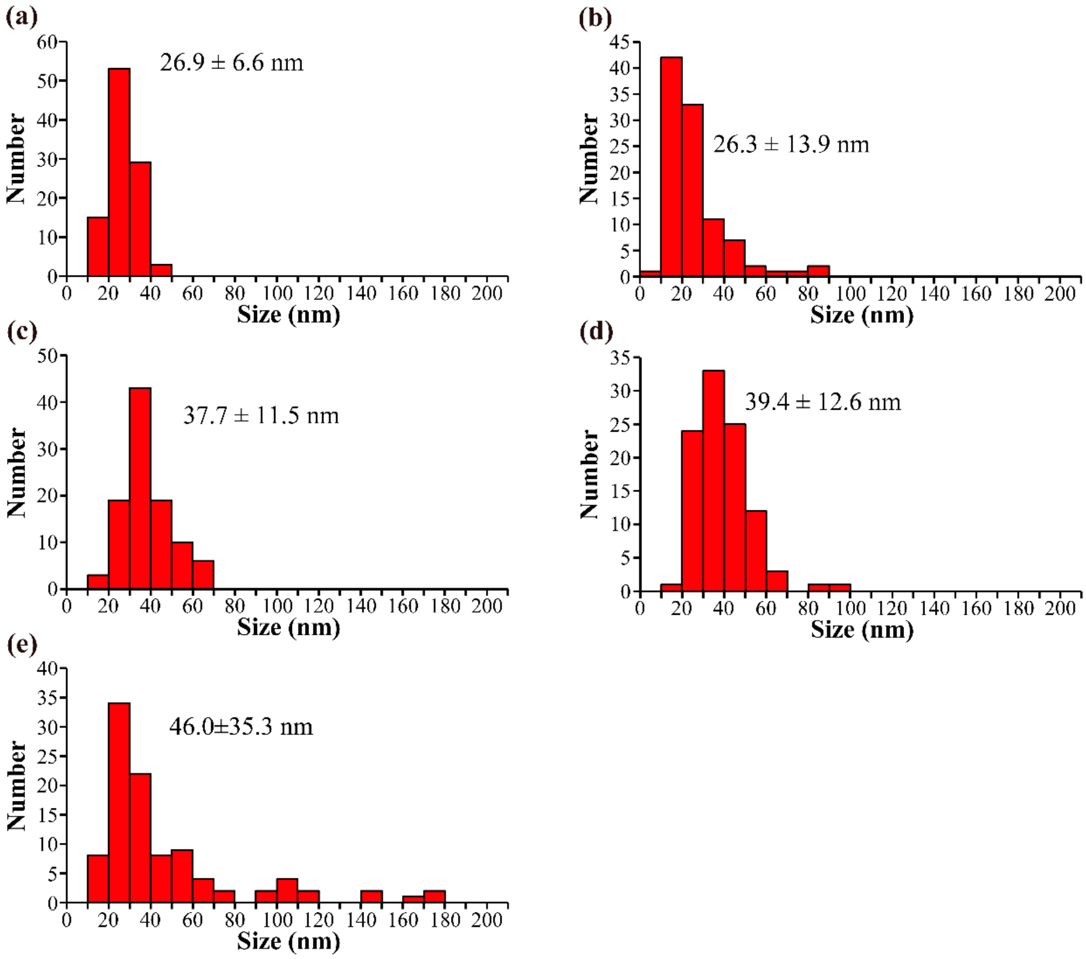

Fig. S10 Particle size histograms of the samples after gold reduction by using [W1 (beads)] = 25 μM with $[HAuCl_4]$ = (a) 0.5 μM, (b) 1 μM, (c) 10 μM, (d) 100 μM and (e) 1000 μM.

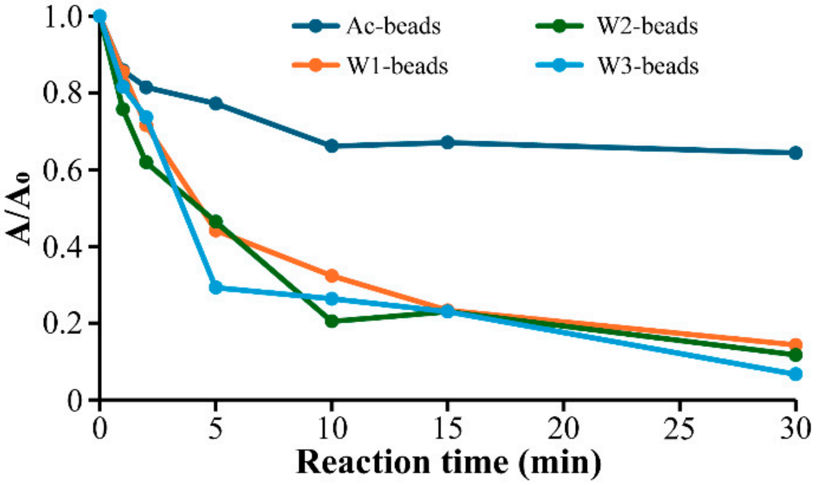

Fig. S11 Catalytic reaction progression of immobilized AuNPs prepared using W1-beads, W2-beads, W3-beads or Ac-Beads. Progression in immobilized AuNPs prepared under various conditions from 0–30 min.

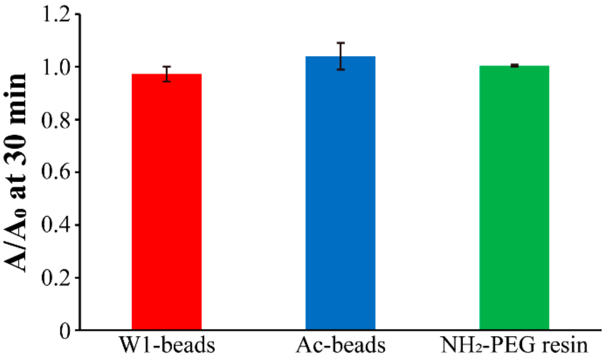

Fig. S12 Progress of 4-nitrophenol reduction after 30 min using the beads without Au ion reduction (before Au ion reduction).

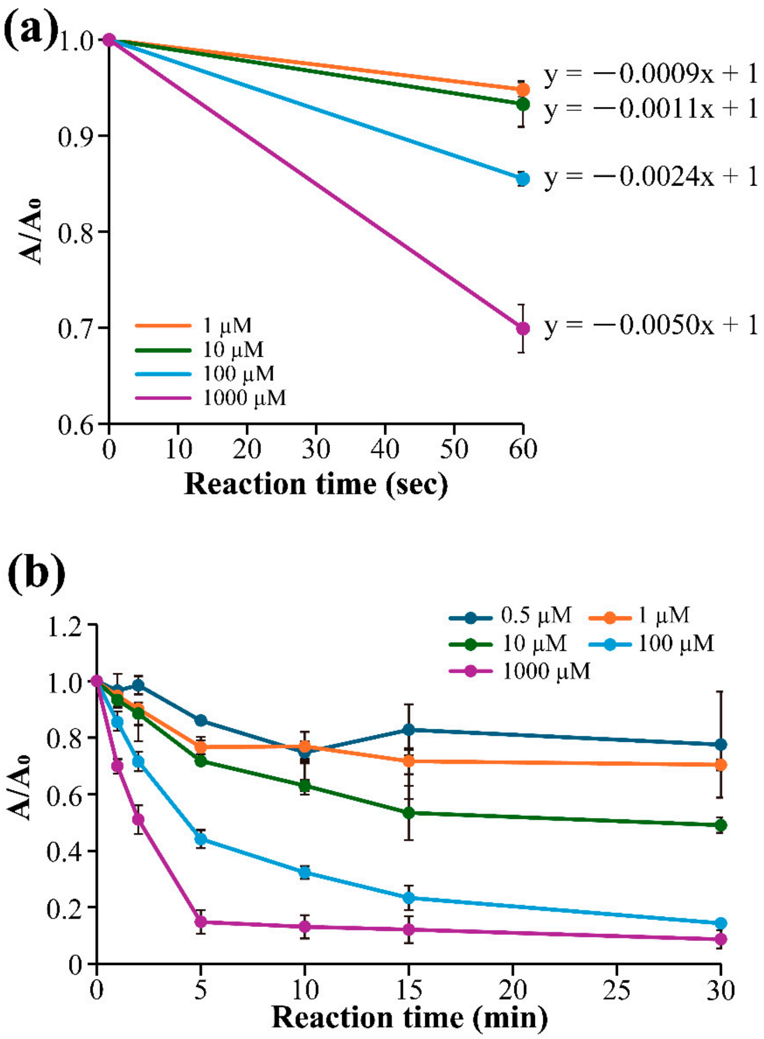

Fig. S13 Catalytic reaction progression of immobilized AuNPs prepared using W1-beads. Progression in immobilized AuNPs prepared under various conditions from (a) 0–60 sec and (b) 0-30 min.

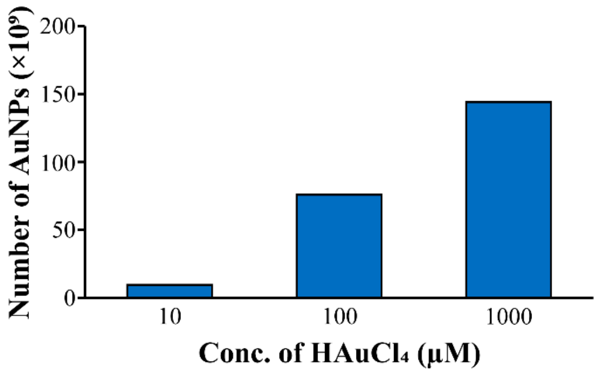

Fig. S14 The number of AuNPs immobilized on peptidyl beads.
